# ^1^H, ^15^N and ^13^C backbone resonance assignments of pentaerythritol tetranitrate reductase from *Enterobacter cloacae* PB2

**DOI:** 10.1007/s12104-017-9791-2

**Published:** 2017-11-22

**Authors:** Andreea I. Iorgu, Nicola J. Baxter, Matthew J. Cliff, Jonathan P. Waltho, Sam Hay, Nigel S. Scrutton

**Affiliations:** 1 Manchester Institute of Biotechnology and School of Chemistry, The University of Manchester, 131 Princess Street, Manchester, M1 7DN UK; 2 Krebs Institute for Biomolecular Research, Department of Molecular Biology and Biotechnology, The University of Sheffield, Firth Court, Western Bank, Sheffield, S10 2TN UK

**Keywords:** Pentaerythritol tetranitrate reductase, Flavoenzyme, Flavin mononucleotide, Backbone resonance assignment, Transverse relaxation optimized spectroscopy

## Abstract

Pentaerythritol tetranitrate reductase (PETNR) is a flavoenzyme possessing a broad substrate specificity and is a member of the Old Yellow Enzyme family of oxidoreductases. As well as having high potential as an industrial biocatalyst, PETNR is an excellent model system for studying hydrogen transfer reactions. Mechanistic studies performed with PETNR using stopped-flow methods have shown that tunneling contributes towards hydride transfer from the NAD(P)H coenzyme to the flavin mononucleotide (FMN) cofactor and fast protein dynamics have been inferred to facilitate this catalytic step. Herein, we report the near-complete ^1^H, ^15^N and ^13^C backbone resonance assignments of PETNR in a stoichiometric complex with the FMN cofactor in its native oxidized form, which were obtained using heteronuclear multidimensional NMR spectroscopy. A total of 97% of all backbone resonances were assigned, with 333 out of a possible 344 residues assigned in the ^1^H–^15^N TROSY spectrum. This is the first report of an NMR structural study of a flavoenzyme from the Old Yellow Enzyme family and it lays the foundation for future investigations of functional dynamics in hydride transfer catalytic mechanism.

## Biological context

Pentaerythritol tetranitrate reductase (PETNR, EC 1.6.99.1) is a monomeric flavoenzyme that possesses a broad substrate specificity and is a member of the Old Yellow Enzyme (OYE) family of oxidoreductases (Toogood et al. 2010). PETNR was originally isolated from an *Enterobacter cloacae* PB2 strain that was thriving on soil contaminated with explosives by exploiting nitrate esters as metabolic nitrogen sources (French et al. 1996). The catalytic cycle of PETNR has been thoroughly investigated using stopped-flow techniques (Khan et al. 2002, 2005; Basran et al. 2003) and it is understood that the reaction chemistry takes place via a bi–bi ping–pong mechanism, with NADPH as the preferred coenzyme (NADH also supports catalysis, but in a less efficient manner). The first catalytic step, described as the reductive half-reaction, consists of the formation of a charge-transfer complex upon NAD(P)H binding, which enables subsequent hydride transfer from the nicotinamide ring C4 atom of NAD(P)H to the N5 atom of oxidized FMN (FMN_ox_). The second step of the reaction, the oxidative half-reaction, is represented by hydride transfer from the N5 atom of reduced FMN to an α,β-unsaturated substrate and, in some cases, additional proton transfer from a solvent molecule. The specific biological role of PETNR is unknown, as for all members of the OYE family of oxidoreductases (Williams and Bruce 2002). However, whilst the biological substrate has yet to be discovered, many studies have unveiled the promiscuous nature of PETNR, as it catalyzes the reduction of a wide variety of α,β-unsaturated compounds, often with high specificity and enantioselectivity towards desired products (Toogood et al. 2011). PETNR acts as an ene-reductase, by reducing 2-cyclohexenone and several steroids to their corresponding alkanes, but also catalyzes the reduction of cyclic triazines, various explosives (e.g. pentaerythritol tetranitrate and trinitrotoluene) and nitrate esters (e.g. nitroglycerin). Several mutagenesis studies have highlighted the versatility of PETNR in the asymmetric reduction of C=C bonds, leading to an increased interest in understanding the physical basis of the catalytic power of this enzyme (Mueller et al. 2010; Fryszkowska et al. 2011; Toogood et al. 2011).

As well as having high potential to be used in biocatalytic processes, PETNR is an excellent model system for studying hydride transfer reactions. Previous kinetic studies have revealed that quantum mechanical tunneling contributes to the enzymatic hydride transfer step from NAD(P)H to the FMN cofactor, as the reaction manifests elevated kinetic isotope effects (Basran et al. 2003). Moreover, fast protein dynamics have been inferred to contribute to catalysis from various temperature and pressure dependence studies, coupled with the use of different isotopic labeling strategies (Pudney et al. 2009, 2013; Longbotham et al. 2016). However, the difference in reactivity of PETNR towards NADPH and NADH is still not clearly understood and the role of fast protein dynamics in catalytic events requires an atomistic description of the enzyme, as kinetic data alone cannot pinpoint or specifically isolate time-resolved structural changes. Herein, we report the near-complete, sequence-specific ^1^H, ^15^N_H_, ^13^C_α_, ^13^C_β_ and ^13^C′ backbone resonance assignments of PETNR in the PETNR:FMN_ox_ complex. The assignments provide a basis for further structural and functional studies by NMR spectroscopy, enabling more complex dynamic studies that can advance our understanding of enzymatic hydride transfer reactions.

## Methods and experiments

All reagents were of analytical grade and were purchased from Sigma-Aldrich (UK), except for ^15^NH_4_Cl and ^2^H_2_O (> 99.9% purity), which were procured from Goss Scientific Ltd. (UK) and ^2^H_7_,^13^C_6_-d-glucose (> 98% purity), which was purchased from Cambridge Isotope Laboratories (USA).

Recombinant PETNR is expressed and purified as a tight non-covalently bound PETNR:FMN_ox_ complex. A pBlueScript II SK(+) plasmid encoding the pONR1 gene was used for PETNR overexpression. The ^2^H,^13^C,^15^N-labeled PETNR:FMN_ox_ complex (40 kDa) was expressed in JM109 *E. coli* cells grown in 100% ^2^H_2_O modified minimal media, using ^15^NH_4_Cl and ^2^H_7_,^13^C_6_-d-glucose as nitrogen and carbon sources, respectively. Cells were incubated at 37 °C with shaking at 200 rpm, until OD_600nm_ = 0.8–1.0, after which the temperature was lowered to 25 °C and PETNR overexpression was induced by the addition of 0.5 mM isopropyl β-d-1-thiogalactopyranoside (IPTG). Cells were grown for a further 24 h before being harvested by centrifugation (6000 rpm for 15 min at 4 °C). All purification steps, which include affinity chromatography using a Mimetic Orange 2 column followed by Source 15Q anion exchange chromatography, were performed at 4 °C, as previously described (French et al. 1996; Barna et al. 2001). A final yield of approximately 70 mg pure PETNR per liter of cell culture was obtained.

Following purification, back exchange to amide protium atoms in the ^2^H,^13^C,^15^N-labeled PETNR:FMN_ox_ complex was promoted by mild destabilization using guanidine hydrochloride (GuHCl) as a denaturing agent, followed by rapid protein refolding. Partial unfolding of PETNR was initiated by mixing the sample (0.25 mM) in a 1:1 ratio with 1.5 M GuHCl solution in 50 mM potassium phosphate buffer (pH 7.0), followed by incubation at room temperature for 120 min. Refolding of PETNR was achieved by rapid 30-fold dilution into 50 mM potassium phosphate buffer (pH 7.0), under vigorous stirring. The resulting sample was filtered using a 0.2 μm filter-syringe to remove any precipitates, buffer-exchanged (to remove GuHCl) and concentrated using a Vivaspin 20 concentrator (10 kDa MWCO, Sigma-Aldrich, UK). The concentration of the PETNR:FMN_ox_ complex was estimated by measuring the FMN-specific absorbance peak at 464 nm (ε = 11.3 mM^−1^ cm^−1^).

All NMR experiments were conducted with samples containing 1 mM ^2^H,^13^C,^15^N-labeled PETNR:FMN_ox_ complex in 50 mM potassium phosphate buffer (pH 7.0) supplemented with 1 mM NaN_3_, 10% (v/v) ^2^H_2_O for the deuterium lock and 0.5% (v/v) trimethylsilyl propanoic acid (TSP) for chemical shift referencing. The samples (300 μL) were centrifuged for 10 min at 13,000 rpm before being transferred to 5-mm Shigemi tubes (Sigma-Aldrich, UK). All NMR experiments were recorded at 298 K on an 800 MHz Bruker Avance III spectrometer running TopSpin version 3.2, equipped with a 5-mm ^1^H/^13^C/^15^N TCI cryoprobe and a Z-field gradient coil. The backbone resonance assignment of PETNR was achieved using standard Bruker ^1^H–^15^N TROSY and TROSY-based 3D HNCA, HNCACB, HN(CO)CACB, HN(CA)CO and HNCO triple resonance experiments (Gardner and Kay 1998). The 3D experiments were acquired using non-uniform sampling with a sine-weighted multidimensional Poisson Gap scheduling strategy (Hyberts et al. 2013). ^1^H chemical shifts were referenced relative to the internal TSP signal, whereas ^15^N and ^13^C chemical shifts were referenced indirectly using nuclei-specific gyromagnetic ratios (Markley et al. 1998). NMR data were processed using TopSpin software version 3.2 and analyzed using CcpNmr Analysis software version 2.4 (Vranken et al. 2005).

## Resonance assignment and data deposition

**Fig. 1:**
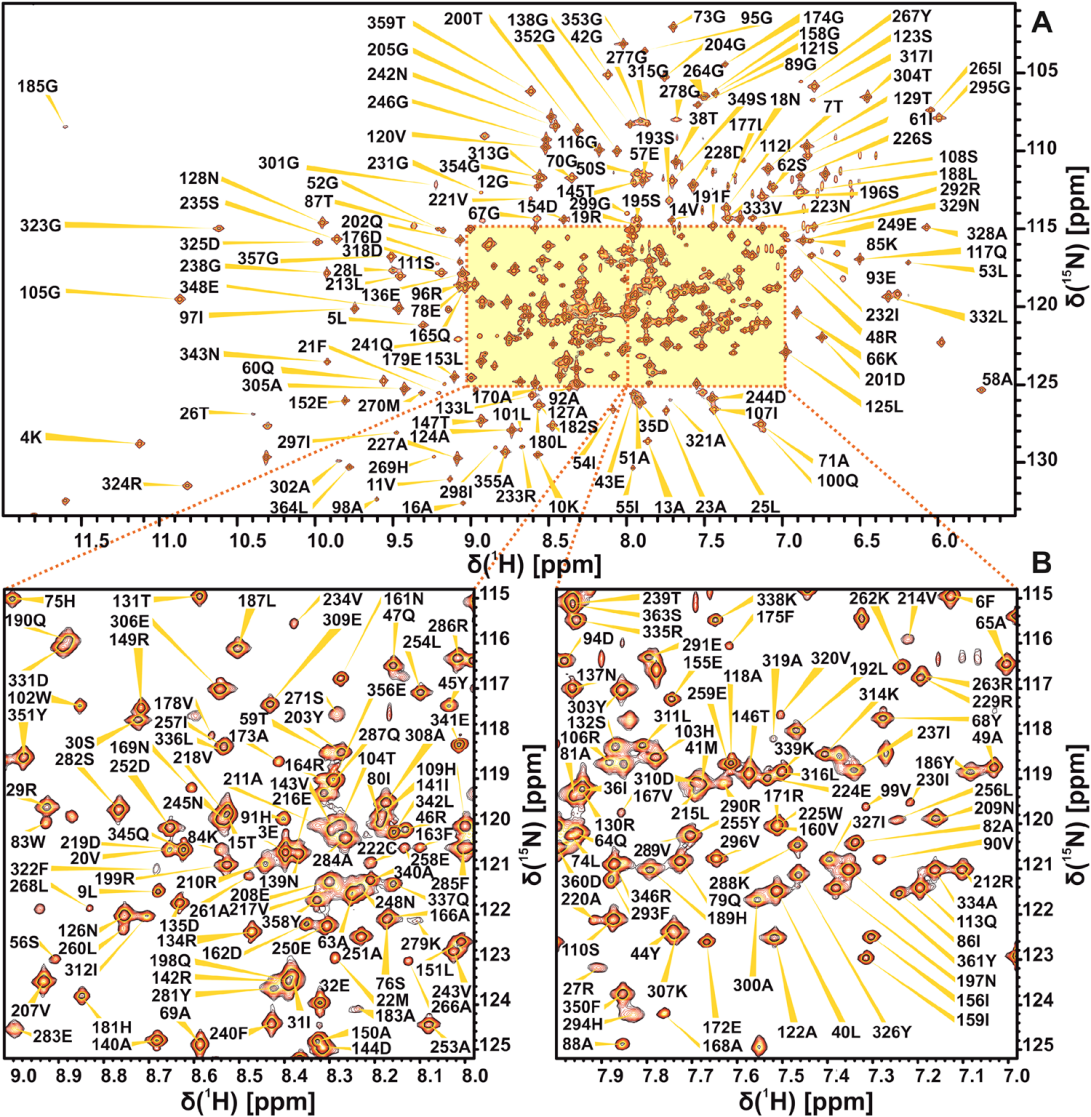
^1^H–^15^N TROSY spectrum of 1 mM uniformly ^2^H,^13^C,^15^N-labeled PETNR:FMN_ox_ complex in 50 mM potassium phosphate buffer (pH 7.0), recorded at 298 K on an 800 MHz spectrometer. The full spectrum (**a**) and two expansions of the crowded regions (**b**) are shown. The assignment of the backbone amide resonances are indicated by sequence number and residue type

Using conventional TROSY-based 3D heteronuclear experiments, sequential backbone assignment of PETNR in the PETNR:FMN_ox_ complex was achieved to a great extent, with 97% of backbone amide groups successfully assigned (333 out of 344 non-proline residues) in the ^1^H–^15^N TROSY spectrum (Fig. 1). A similar degree of assignment (97%) was achieved for the corresponding ^13^C_α_, ^13^C_β_ and ^13^C′ resonances: 354 out of all 364 C_α_ atoms, 320 out of all 330 C_β_ atoms and 354 out of all 364 C′ atoms. The chemical shift assignments have been deposited in the Biological Magnetic Resonance Bank (BMRB: http://www.bmrb.wisc.edu/) under the accession number 27224.

**Fig. 2:**
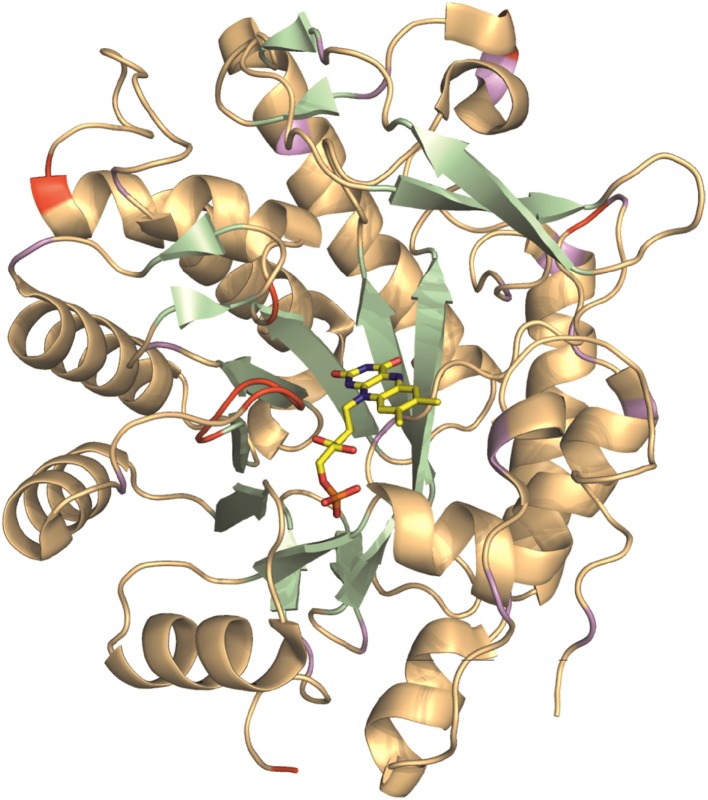
Cartoon representation of the crystal structure of the PETNR:FMN_ox_ complex [PDB: 5LGX (Kwon et al. 2017)], highlighting the extent of backbone amide resonance assignments. Assigned residues are colored light orange (for α-helices and loops) and pale green (for β-strands), with unassigned residues shown in red and proline residues in purple. The non-covalently bound FMN cofactor is depicted as yellow sticks

The assignment extends the list of large molecular systems that have undergone essentially complete backbone assignment, at a time where limits are being pushed with studies of very large biological systems (Sprangers and Kay 2007). The assignment of PETNR was facilitated by the wide dispersion and favorable line shape of the resonances in the ^1^H–^15^N TROSY spectrum, attributed to the globular folded structure, isotopic labeling strategy and correlation time of the complex (21 ns). There are ten residues that remain unassigned in the ^1^H–^15^N TROSY spectrum (A2, G34, G115, H184, S206, E272, T273, D274, L275 and A276), which are mainly located in a mobile loop (E272–Y281), at the N-terminus and in other solvent-exposed regions of PETNR (Fig. 2). The E272–Y281 loop is located at the edge of the substrate-binding pocket of the active site, but is solvent exposed in the PETNR:FMN_ox_ complex and residues T273–A284 have high temperature factors and limited secondary structure, as it has been observed from crystallographic data (Barna et al. 2001). Thus, both conformational exchange and solvent exchange are probably the source of signal attenuation beyond detection in the ^1^H–^15^N TROSY spectrum for residues E272, T273, D274, L275 and A276. Residue A2, located at the N-terminus, and residues G34, G115 and S206, located at the surface of PETNR, could not be assigned since their ^1^H–^15^N TROSY correlations are likely attenuated due to fast exchange with solvent. Residue H184 is known to be involved in substrate coordination (Barna et al. 2001) and, in the absence of bound substrate, will probably be undergoing conformational exchange on the millisecond timescale.

**Fig. 3:**
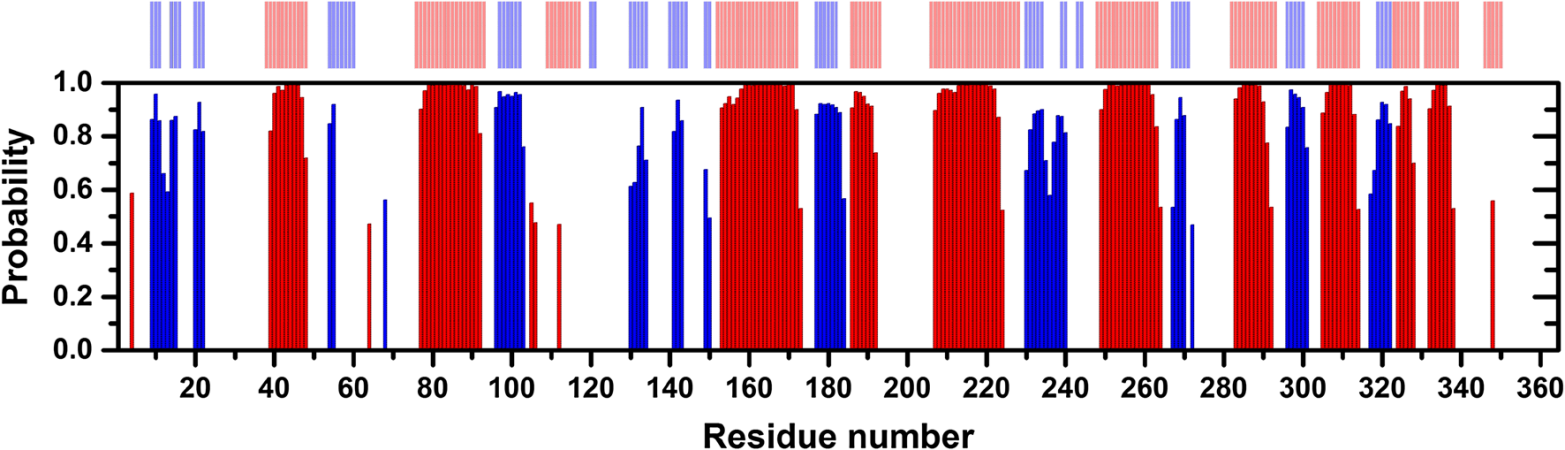
Backbone secondary structure prediction of PETNR in the PETNR:FMN_ox_ complex obtained with TALOS-N software (Shen and Bax 2013) using the backbone ^1^H_N_, ^15^N, ^13^C_α_^13^C_β_ and ^13^C′ chemical shifts. The secondary structure prediction is depicted as red bars and blue bars for α-helices and β-strands, respectively, with the height of the bars reflecting the probability of each element, as assigned by the software. As a comparison, the secondary structure observed in the crystal form of the PETNR:FMN_ox_ complex [PDB: 5LGX (Kwon et al. 2017)] is shown at the top of the figure in the same color representation

An empirical prediction of the secondary structure elements of PETNR in the PETNR:FMN_ox_ complex was performed by uploading the backbone ^1^H_N_, ^15^N, ^13^C_α_, ^13^C_β_ and ^13^C′ chemical shift assignments to the TALOS-N webserver (Shen and Bax 2013). The results of the prediction are illustrated in Fig. 3, along with a comparison of the secondary structure present in the crystal form of the complex. The prediction derived from the NMR data is in very good agreement with the crystallographic data, with all the specific elements of the eight-stranded α/β barrel (TIM barrel) predicted accurately. The results provide high confidence in the assignments of the PETNR:FMN_ox_ complex.
